# Atmospheric Chemistry and Air Pollution

**DOI:** 10.1100/tsw.2003.18

**Published:** 2003-04-07

**Authors:** Jeffrey S. Gaffney, Nancy A. Marley

**Affiliations:** Environmental Research Division, Argonne National Laboratory, 9700 South Cass Avenue, Building 203, Argonne, IL 60439-4843, USA

**Keywords:** ozone, peroxyacetyl nitrate, volatile hydrocarbon, aerosol, troposphere, chlorine radical, hydroxyl radical, peroxy radicals, acid rain, radiative balance, alternative fuel

## Abstract

Atmospheric chemistry is an important discipline for understanding air pollution and its impacts. This mini-review gives a brief history of air pollution and presents an overview of some of the basic photochemistry involved in the production of ozone and other oxidants in the atmosphere. Urban air quality issues are reviewed with a specific focus on ozone and other oxidants, primary and secondary aerosols, alternative fuels, and the potential for chlorine releases to amplify oxidant chemistry in industrial areas. Regional air pollution issues such as acid rain, long-range transport of aerosols and visibility loss, and the connections of aerosols to ozone and peroxyacetyl nitrate chemistry are examined. Finally, the potential impacts of air pollutants on the global-scale radiative balances of gases and aerosols are discussed briefly.

### INTRODUCTION

Humans are benthic organisms living in a sea of air. That air is made up primarily of nitrogen (78.1 %), oxygen (20.9%), and argon (0.9%). The rest of the atmosphere is composed of water, carbon dioxide, and a large number of trace gases. Many of the organisms on the planet directly affect the chemistry of the atmosphere. Plants take up carbon dioxide and transform it into organic diacids, then into cellular materials. Plants and animals use oxygen in the air for oxidative biochemical reactions that release heat and energy during respiration and reintroduce carbon dioxide and many trace gas species. Atmospheric chemistry is a complicated subject that addresses gas-, liquid-, solid-, and mixed-phase chemical reactions in our atmosphere and focuses on the trace gas species. Like the trace molecules in our bodies that enable us to live, the trace gases and aerosols in our atmosphere maintain a very crucial part of the planet — its atmosphere — without which we could not survive.

Our atmosphere is composed of a number of important regions that are defined by thermal structure as a function of altitude[1,2]. As altitude increases, air pressure drops off rapidly (see Fig. 1). The temperature structure of the atmosphere is somewhat complex because of exothermic chemical reactions in the upper atmosphere that are caused by the absorption of high-energy photons (ultraviolet-C and ultraviolet-B) from incoming solar radiation (see Fig. 1). The breaking of molecular bonds produces atomic species and results in recombination reactions that form ozone ($O_3$) and other important gases that can filter the incoming ultraviolet (B and C) light and act as a protective optical shield against harmful solar radiation. Most of this shielding occurs in the stratosphere, where a great deal of recent interest has been focused because of the depletion of the stratospheric ozone layer by anthropogenic use of chlorofluorocarbons[1]. As altitude increases from sea level, temperature in the troposphere decreases up to about 12–15 km — at the tropopause — where temperature begins to increase in the stratosphere. The troposphere, where we live, is where we focus this mini-review.

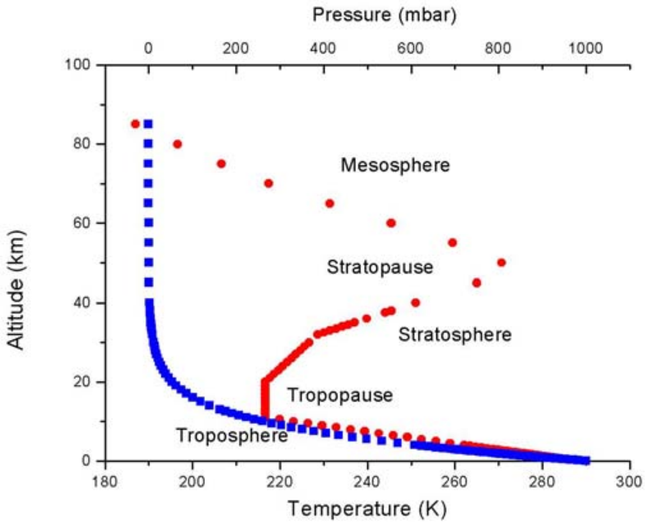

**FIGURE 1**. Pressure (mbar, blue ■) and temperature (K, red ●) profiles for Earth's atmosphere (1976 standard atmosphere calculation).

In the troposphere, humans have begun to alter the chemistry of the air through the emission of tremendous amounts of carbon dioxide ($CO_2$), carbon monoxide (CO), trace organics, and sulfur and nitrogen oxides from a wide variety of human activities. The release of gases and aerosols from the combustion of fossil fuels and other sources affects the quality of tropospheric air (which we breathe) directly or indirectly via chemical reactions that form secondary pollutants. Pollutants can result in human health effects, crop losses, visibility reduction, urban heat island effects, regional weather modification, and global climate change.

In this paper we highlight key issues related to the chemistry of the troposphere and the quality of the air. We give a brief history of air pollution, with particular attention to photochemical air pollution and its basis, and we explore urban, regional, and global impacts of air pollutants, particularly with regard to the use of fossil and alternative fuels.

## BRIEF HISTORY OF AIR POLLUTION

### Early Air Pollution

Air pollution has long been recognized as a major problem affecting cities. Moses Maimonedes (1135–1204) was a noted jurist, physician, and rabbi who was born in Spain and traveled to Cairo, Egypt. There he wrote about the quality of the air in the cities where wood burning was used as the source of energy [1]:

> *Comparing the air of cities to the air of deserts and arid lands is like comparing waters that are befouled and turbid to waters that are fine and pure. In the city, because of the height of its buildings, the narrowness of its streets, and all that pours forth from its inhabitants and their superfluities . . . the air becomes stagnant, turbid, thick, misty, and foggy. . . . If there is no choice in this matter, for we have grown up in the cities and have become accustomed to them, you should . . . select from the cities one of open horizons . . . endeavor at least to dwell at the outskirts of the city. . . .*
>
> *If the air is altered ever so slightly, the state of the Psychic Spirit will be altered perceptibly. Therefore you find many men in whom you can notice defects in the actions of the psyche with the spoilage of the air, namely, that they develop dullness of understanding, failure of intelligence, and defect of memory. . . .*

By the 16th century, coal of high sulfur content was being burned in many European cities, particularly in London. After the Restoration, John Evelyn, Esquire (1620–1706), wrote *Fumifugium*[3], a text concerning the air pollution that was plaguing London at that time and continued to do so into the 20th century. London was typically lost in a cloud of smoke from industrial combustion of wood and dirty coal. Evelyn wrote one of his best-known works, *Fumifugium*, to bring attention to the air quality issue and also to give the king and Parliament some possible solutions to consider. Evelyn's writings were presented to Charles II, on whose command they were published soon after, in 1661, along with an act for Parliament to consider (though no act was ever passed). In *Fumifugium* Evelyn wrote:

> *This horrid Smoake which obscures our Church and makes our Palaces look old, which fouls our Cloth and corrupts the Waters, so as the very Rain, and refreshing Dews which fall in the several seasons, precipitate to impure vapour, which, with its black and tenacious quality, spots and contaminates whatever is exposed to it. . . . It is evident to every one who looks on the yearly Bill of Mortality, that near half the children that are born in London die under two years of age.*

In contrast, children born and raised in the rural areas were likely to live to 40 years of age.

### Killer Smog in London, England, and Donora, Pennsylvania, in the 1950s

During the 1950s this same combination of high-sulfur coal and ash led to the so-called *killer smogs* in London, England, and in Donora, Pennsylvania. Meteorological conditions (thermal inversions) trapped pollution produced by local industrial use of high-sulfur coal and led to the deaths of thousands and the subsequent passing of the Clean Air Act in Britain. These fatal events occurred during foggy, low-inversion events in which smoke and fog combined to become *smog*.

### Photochemical Smog in the Los Angeles Air Basin in the 20th Century

A different type of smog was observed for the first time in the 20th century. The Los Angeles air basin in southern California was known during the early 1940s for its *photochemical smog* produced by absorption of light by gases in the air[1,2,4]. In the late 1940s and early 1950s, farmers began to note serious damage to some crops, especially to romaine lettuce and parsley in the San Gabriel Valley. Rubber products were found to deteriorate at an accelerated rate. Various oxidants were suspected.

John Middleton, a professor of plant pathology at the University of California, and his research group first noted that sulfur dioxide and hydrofluoric acid were not the cause of the damage, but that oxidation, not reduction, was at hand[5,6]. As Edgar Stephens pointed out in his paper on smog studies of the 1950s[4], "the state of the art of analytical chemistry in 1945 was inadequate to deal with the problem." The problem was to identify the key oxidants causing the damage with the analytical tools available at that time.

A perfumery chemist at the California Institute of Technology in Pasadena, Arie J. Haagen-Smit, noted that smog smelled like his terpene laboratory. Ellis Darley of the Citrus Experiment Station at the University of California, Riverside (UCR), began to expose plants with a fumigation system to a wide variety of chemicals, attempting, with little success, to determine the cause of plant bronzing. In 1950, Haagen-Smit fumigated a plant under laboratory conditions with the products from the ozonolysis of gasoline vapors and was able to reproduce the plant pathology[7]. His theory was that partially oxidized hydrocarbons produced by reaction with ozone were the cause of the plant symptoms.

Haagen-Smit followed these studies with photochemical investigations using nitrogen dioxide ($NO_2$) and hydrocarbons (known to be emitted in vehicle exhaust), in which he demonstrated that their photolysis could produce the oxidant ozone. He also showed that the reaction mixture would lead to the plant and rubber damage observed in the Los Angeles air basin[8,9]. Other photochemical smog symptoms included eye irritation and aerosol production leading to the characteristic brown haze observed in the atmospheric boundary layer. The demonstration that olefins (alkenes) are more reactive than other hydrocarbons in producing smog symptoms led to studies to identify the sources of these emissions in the Los Angeles area. The two likely sources were automobiles and oil refineries.

### Peroxyacetyl Nitrate and the Mechanisms of Smog Production

The real questions that arose from the Haagen-Smit studies concerned the identities of the actual products and the mechanism of smog production. Was ozone the cause, or were other reactive gases responsible for the plant damage and eye irritation? These questions and others posed by the chemists of the time initiated the study of air pollution chemistry and the application of optical spectroscopy to the problem at hand. Edgar Stephens, Philip Hanst, Robert Doerr, and William Scott from the Franklin Institute Laboratories in Philadelphia used the first long-path infrared gas cell to identify some of the products. This instrument, constructed in 1954, enabled probing of the infrared spectrum of photolyzed mixtures of olefins and nitrogen oxides[10,11] and the discovery of a second key oxidant in photochemical smog, peroxyacetyl nitrate ($CH_3CO$-OO-$NO_2$).

Initially, peroxyacetyl nitrate was discovered during investigation of the photochemical oxidation of 3-methylpentane in the presence of nitrogen dioxide. A very interesting, unexpected set of infrared bands was observed, and further studies with biacetyl found the same strong absorptions[11]. The strong bands at 1740 and 1841 $cm^{-1}$ were quite unique, and the molecule responsible was labeled "Compound X." The Franklin Institute research team attempted to isolate Compound X from the biacetyl reaction, but a two-drop sample trap exploded violently before they were able to obtain a mass spectrum. The five structures suggested all proved to be incorrect. The correct structure, $CH_3CO\text{-}OO\text{-}NO_2$, was not really determined until the leading candidate, acetyl nitrate, was synthesized by Edward Schuck and George Doyle and their results were presented in 1959[12]. This work showed similarities with the unknown nitrate but also proved that acetyl nitrate was not Compound X.

In 1961, Philip Leighton's classic textbook, *Photochemistry of Air Pollution*[13] summarized what was known about the complex series of chemical reactions involved in the production of ozone and also initiated a series of events that finally led Edgar Stephens to produce the correct structure for Compound X: peroxyacetyl nitrate. This structure was accepted as correct and finally confirmed in 1974 by use of the isotopically labeled compound in infrared studies[14].

Further studies initiated in 1958 identified peroxyacetyl nitrate as a key compound causing the bronzing of leaves of petunias and other susceptible plants. These studies, done with O. Clifton Taylor of the UCR Horticulture Department[15], led to the use of gas chromatographic separation of peroxyacetyl nitrate from the photochemical reaction mixture[16,17]. Further characterization showed that the compound is a potent eye irritant. The collaboration between chemists and plant pathologists led to the observation that peroxyacetyl nitrate and some of its analogs are 10–50 times more toxic to plants than ozone. Indeed, peroxyacetyl nitrate, hydrogen peroxide, and nitrogen dioxide were identified in the first air pollution criteria documents, but they were later omitted from the Clean Air Act of 1970 because of difficulties in synthesizing and measuring standards at that time. This important collaboration at the UCR Statewide Air Pollution Research Center was one of the first to begin interdisciplinary work on air quality problems and to demonstrate the value of and need for a collaborative approach.

### PHOTOCHEMISTRY — SOME BASICS

Photochemical smog has now been observed in every major city on the planet, and the release of nitrogen oxides and hydrocarbons into the air has been found to lead to the photochemical production of ozone, peroxyacyl nitrates, and a variety of secondary species including aerosols. Here we present some basics about photochemistry to set the stage for a review of the processes that generate photochemical smog.

In photochemical reactions, the absorption of light by molecules leads to the dissociation of chemical bonds. Thus, three things are needed for a photochemical reaction to occur. You must have a molecule, that molecule must have absorption bands, and the molecule must be in the presence of a light source with appropriate energy to be absorbed by the molecule. Leighton's classic textbook *Photochemistry of Air Pollution*[13] describes in detail the photolysis of key air pollutants, leading to photodissociation and subsequent free-radical chain reactions that culminate in smog formation.

We discuss this subject only briefly here, but we refer the reader to a number of classic textbooks on the subject of photochemistry (inorganic and organic), as well as spectroscopy in general[18,19,20,21,22,23,24,25,26].

#### Properties of Light

Light behaves like a wave and like a particle. Considered as a particle, light is referred to as an energy unit or quantum with no mass. The energy of a quantum of light is given by this familiar equation:

$$E = h\nu = hc/\lambda \quad (1)$$

Here E is the energy in ergs, h is Planck's constant, c is the speed of light in a vacuum ($2.998 \times 10^8$ m $s^{-1}$), and $\nu$ and $\lambda$ are the frequency and wavelength, respectively. The first law of photochemistry (Grotthus-Draper law) states that only the light absorbed by a molecule can be effective in producing photochemical change in the molecule. Therefore, one must know the absorption spectra of the molecules, their intermediates, and their products to understand photochemical mechanisms. The strength of a molecule's absorption is given by the well-known Beer-Lambert relationship:

$$\log (I_o/I) = \varepsilon Cl \quad (2)$$

Here $I_o$ is the intensity of light incident on the molecules at the front of a column of molecules, I is the light intensity at the back of the column of molecules, C is the concentration of molecules in the column, l is the length of the column, and $\varepsilon$ is the extinction coefficient in appropriate units (usually L mole $cm^{-1}$ for solutions and $atm^{-1}$ $cm^{-1}$ at a specified temperature for gases).

### Absorption of Light by Molecules

Molecules that absorb radiation of sufficient energy can become electronically excited. If the electronic state of the molecule has a pathway that allows dissociation of a molecular bond, this is a *primary photochemical* proces*s*. Radiation at a sufficient energy to cause chemical reactions for the molecule in question is called *actinic* radiation. If the excitation leads to a radiative process such as fluorescence or phosphorescence that returns the molecule to its ground state without chemical change, the process is considered *photophysical.* Some photophysical processes, such as collisional deactivation and energy transfer to other molecules, can lead to chemical changes. However, these are *secondary photochemical* processes, because the molecules undergoing the chemical change are typically not the light-absorbing species[1,18].

The *quantum yields* of the photochemical and photophysical processes are given by the number of electronically excited molecules that proceed down a particular photochemical pathway relative to the total number of photons absorbed. Quantum yields are usually given at the specific wavelengths of the radiation absorbed. The maximum quantum yield for a molecule is unity, and it would involve a single process. For example, the quantum yield for the photolysis of nitrogen dioxide at a wavelength of 300 nm is 1. This means that for every photon absorbed by nitrogen dioxide at 300 nm, 1 molecule of nitric oxide (NO) and 1 ground-state oxygen atom (O) are produced. For other molecules (aldehydes, ketones, etc.), more than a single pathway can be observed, and quantum yields are always <1. In many cases the quantum yield is quite dependent on the energy of the photons and therefore the wavelength of the incident light. The result is a quantum yield that varies as a function of wavelength or frequency.

In some cases, *radical species* (which have one or more unpaired electrons) can be produced by photolysis. In the presence of another reactant, the radical species can cause a chain reaction. In these cases the quantum yield for the production of the radical may appear to be >1. However, this observation indicates that a chain reaction is occurring, because primary processes can never exceed a quantum yield of 1.

Types of photochemical reactions that can occur are photodissociation, intramolecular rearrangements, isomerization reactions (cis-trans rearrangements), hydrogen atom abstraction reactions, photodimerization, and photosensitized reactions (collisional energy transfer).

### Oxygen and Ozone in the Troposphere

In Earth's troposphere, the sun is the light source for the important photochemical reactions. The radiation reaching the troposphere is strongly filtered by molecular oxygen ($O_2$) and ozone that is produced in the stratosphere by oxygen photolysis, known as the Chapman cycle[1,2]:

$$O_2 + h\nu \rightarrow 2\ O \qquad (3)$$

$$O + O_2 + M \rightarrow O_3 + M \qquad (4)$$

$$O + O_3 \rightarrow 2\ O_2 \qquad (5)$$

In reaction 4, M is a third body that allows formation of ozone by collisional release of the energy produced from ozone formation. This three-body reaction therefore is pressure dependent. M can be nitrogen, oxygen, or argon.

Oxygen and ozone filter out harmful ultraviolet radiation, so that incoming tropospheric radiation is ≥290 nm in wavelength. This incoming light is what is available for photochemical processes in the troposphere. Typically, the light at wavelengths below 400 nm is of an energy that can allow photodissociation of key tropospheric species. This radiation has been referred to as *actinic ultraviolet* by a number of researchers.

### Light Scattering

Light can also be scattered by molecules in the atmosphere and by aerosols or particles and cloud droplets that are present. The intensity of sky radiation or scattered light in the atmosphere is difficult to measure because of the variability of particle concentrations, chemical composition, and size distributions. Reflection of light from Earth's surface can also affect photon availability for photochemical reactions.

### Light Reflection and Albedo

Most surfaces reflect some portion of total incident radiation. Black surfaces absorb practically all of the light, while white surfaces are highly reflective. A totally reflective surface would have an *albedo* (fraction of incident light that is reflected) of 1. The albedos of different surfaces range from 0.05 for some forests to 0.85 for some snow surfaces[13,27]. Indeed, clouds are important as scatterers, and low-lying clouds, with an apparent albedo of 0.7–0.95[27], can act as important reflectors of incoming radiation. Readers interested in further discussion of this subject are referred to Leighton and to Finlayson-Pitts and Pitts[1,2,13]. For the best estimates of actinic flux at Earth's surface as a function of wavelength and solar zenith angle, the reader is referred to work by Peterson and by Demerjian et al.[28,29], as well as to an overview by Finlayson-Pitts and Pitts[1].

### Pollutants of Concern

The key atmospheric pollutants of concern are ozone, nitrogen dioxide, formaldehyde (and higher analogs), conjugated ketones (unsaturated), nitrogen trioxide, nitrous acid, and alkyl nitrites in the gas phase[1,2,13]. In aqueous and aerosol phases, photolytically important species include nitrate[1,2,30]. In reviewing some of the key photochemical species, we note that many other possible organic photochemical reactions might also be important in the chemistry of the troposphere, particularly on aerosol surfaces.

### Ozone

The photolysis of ozone is of major importance in the chemistry of the troposphere, because at wavelengths of 290–336 nm this reaction yields *electronically excited oxygen* atoms:

$$O_3 + h\nu \rightarrow O_2 + O\ (^1D) \tag{6}$$

The quantum yields for this process are close to 1 (approximately 0.95), with some *ground-state oxygen* atoms, O ($^3P$), being formed in competing processes[31,32,33]. Photolysis of ozone is extremely important, because the reaction of O ($^1D$) with water vapor ($H_2O$) is extremely fast and produces hydroxyl radical (OH)[1,2,31]:

$$O\ (^1D) + H_2O \rightarrow 2\ OH \tag{7}$$

As we will see in subsequent sections of this review, the reactions of hydroxyl radical are key to the formation of ozone in air pollution events and also to the removal of many organics from the atmosphere. In this regard, hydroxyl radical acts as a cleansing agent.

### Nitrogen Dioxide

Another important species for the photochemistry of the troposphere is nitrogen dioxide. The removal of ultraviolet radiation of very high energy by stratospheric absorption of oxygen and ozone means that the solar radiation reaching the troposphere has insufficient energy to break the O–O bond. Therefore, oxygen cannot be photolyzed in the troposphere to produce oxygen atoms and ozone. Nitrogen dioxide, however, can be photolyzed in the region of 290–423 nm[2,31]:

$$NO_2 + h\nu\ (290\text{–}423\ nm) \rightarrow NO + O\ (^3P) \tag{8}$$

Subsequent reaction of the ground-state oxygen atom with molecular oxygen can produce ozone in the troposphere:

$$O\ (^3P) + O_2 + M \rightarrow O_3 + M \tag{9}$$

As in the formation of ozone in the troposphere (equation 4), M is a third body. Quantum yields for the photolysis are ≥0.95 at 290–394 nm, 0.695 at 400 nm, 0.130 at 410 nm, and 0.004 at 423 nm[2,31].

### Nitrous Acid

Another important photochemical reactant is nitrous acid (HONO), which has a number of strong absorption bands in the wavelength region 300–400 nm[2,34,35]. Nitrous acid photolyzes in this region to produce hydroxyl radical:

$$HONO + h\nu\ (310\text{–}400\ nm) \rightarrow OH + NO \tag{10}$$

The quantum yield for this reaction is believed to be 1 in the region 300–400 nm[2,36]. This photochemical reaction can also play an important role, particularly in urban air masses, as it is a source of nitric oxide and hydroxyl radical, both key players in the tropospheric formation of ozone.

### Nitrate Radical

Ozone and nitrogen dioxide can react with each other to form nitrate radical ($NO_3$):

$$O_3 + NO_2 + M \rightarrow NO_3 + M \quad (11)$$

Nitrate radical is a very reactive species in nighttime processes, as we will see later. It is unusual in that it absorbs strongly in the red region of the visible spectrum (620–670 nm)[2,37,38]. There are two possible pathways for the photolytic dissociation of nitrate radical:

$$NO_3 + h\nu \rightarrow NO_2 + O\ (^3P) \quad (12)$$

$$NO_3 + h\nu \rightarrow NO + O_2 \quad (13)$$

The photolysis actually proceeds via pathway 12, with a quantum yield of 1 at 570–585 nm[2,39,40,41]. Thus, the photolysis produces an oxygen atom that rapidly forms ozone in the troposphere, leading to the regeneration of the starting reactants, ozone and nitrogen dioxide. Because this photolysis is in the visible region and large numbers of photons of visible light hit the surface, photolysis rate constants for reaction 12 at Earth's surface are estimated to be 0.17–0.19 $s^{-1}$[41].

### Hydrogen Peroxide

Hydrogen peroxide ($H_2O_2$) has weak absorption band tails of relevance to tropospheric photochemistry at 290–350 nm. Hydrogen peroxide photolyzes to form two hydroxyl radicals:

$$H_2O_2 + h\nu \rightarrow 2\ OH \quad (14)$$

The quantum yield for this photolysis is usually taken as 1 at 290–350 nm[2].

### Organic Species

Organic molecules can also undergo photochemical reactions. Like nitrous acid, alkyl nitrites (RONO) undergo photolytic dissociations to form alkoxy radicals (RO) and nitric oxide:

$$RONO + h\nu \rightarrow RO + NO \quad (15)$$

These processes are so fast that the alkyl nitrites are not readily observed in the atmosphere[2,31].

Probably the best-studied organic photochemical reactions are those of the simple aldehydes, particularly formaldehyde (HCHO)[1,2,13,18]. Formaldehyde is known to photolyze in the tropospherically important wavelength region of 290–356 nm via two pathways:

$$HCHO + h\nu \rightarrow HCO + H \quad (16)$$

$$HCHO + h\nu \rightarrow H_2 + CO \quad (17)$$

One pathway forms the formyl radical (HCO), which is discussed below in the section "Volatile Organics in Urban Air". The other pathway forms a hydrogen atom (H), which can combine with molecular oxygen in the atmosphere to form the hydroperoxyl radical ($HO_2$). Along with its organic counterparts, the alkyl peroxy radicals ($RO_2$), the hydroperoxyl radical plays an important role in the oxidation of nitric oxide in polluted air. In the region of interest, 290–356 nm, the quantum yields for reaction 16 are 0.78 at 300 nm, 0.62 at 319 nm, 0.26 at 331 nm, and 0 at 346 nm. Pathway 17 has quantum yields of 0.21 at 300 nm, 0.37 at 319 nm, 0.697 at 331 nm, 0.47 at 346 nm, and 0.197 at 356 nm[2,42].

For acetaldehyde ($CH_3CHO$), three pathways for photolytic reaction are possible:

$$CH_3CHO + h\nu \rightarrow CH_3 + HCO \quad (18)$$

$$CH_3CHO + h\nu \rightarrow CH_4 + CO \quad (19)$$

$$CH_3CHO + h\nu \rightarrow CH_3CO + H \quad (20)$$

Reaction 20 typically accounts for less than 1% of the overall photolytic processes at wavelengths above 290 nm[2,42]. At 290 nm, reaction 18 has a quantum yield of 0.53, while that of reaction 19 is 0.01. At 300 nm, the quantum yield is 0.43 for pathway 18 and negligible for the other pathways. The quantum yield for reaction pathway 18 drops off to 0.17 at 315 nm and is essentially zero at 330 nm, showing a very strong dependence on the energy of the photons. The two products from photolysis pathway 18 form methylperoxy radical ($CH_3O_2$), carbon monoxide, and hydroperoxyl radical in the presence of oxygen in the troposphere. The peroxy radicals play important roles in the oxidation of nitric oxide in the troposphere that will be discussed in detail in the next section.

Higher-analog ketones and aldehydes can also photolyze in the troposphere, but they are not considered here. The reader is referred to works that discuss these processes in detail[18,19,20,21,22,23,24,25,26,42].

### URBAN AIR POLLUTION

As noted above in the section "Brief History of Air Pollution", urban air pollution consists of a number of key species, including both primary and secondary pollutants, in gas, liquid, and particulate phases. The primary pollutants are emitted directly into the troposphere from various anthropogenic sources, principally related to energy production (combustion of fossil fuels). The primary pollutants include carbon monoxide, nitric oxide and some nitrogen dioxide, sulfur dioxide, volatile hydrocarbons, carbonaceous soots, and a variety of trace constituents including aldehydes and trace metals. Primary emissions of nitric oxide and nitrogen dioxide emissions are typically referred to as $NO_x$, where $NO + NO_2 = NO_x$.

Secondary pollutants are produced from primary pollutants by chemical reactions occurring in the presence of oxygen in the troposphere. These processes are primarily photochemical reactions and radical reactions initiated by photochemical processes mentioned in the previous section. These chemical transformations include the oxidation of $NO_x$ to form nitric acid, organic nitrates, and peroxyacyl nitrates. The oxidation of nitric oxide to form nitrogen dioxide, the production of ozone by the photolysis of nitrogen dioxide, and the formation of sulfuric acid from sulfur dioxide are further examples of production processes for secondary pollutants.

Here we address the secondary transformation chemistries that were first observed in urban air, beginning with the photochemical production of ozone in an urban environment. Note that these same basic chemical reactions occur on regional and global scales but at slower rates.

### Ozone and Other Oxidants in Urban Air

#### *Ozone*

Ozone was one of the first secondary pollutants to be observed in urban air sheds. Los Angeles was well known for its photochemical smog and the associated high ozone levels as early as the 1950s. The unanswered question concerned the mechanism leading to the high levels of ozone produced in this and other urban centers. Haagen-Smit's work clearly indicated that volatile hydrocarbons and nitrogen oxides are at the heart of the process. In Los Angeles the obvious sources for these two types of pollutants were automobiles, local refineries, and stationary power plants.

#### *Nitrogen Oxides*

Automobiles are now known to be major sources of nitric oxide and nitrogen dioxide ($NO_x$). The internal combustion engine operates at high temperatures and pressures. Under these conditions, air (nitrogen and oxygen) reacts efficiently to form $NO_x$, principally nitric oxide. Nitric oxide is known to react with oxygen in the atmosphere to form nitrogen dioxide by the following reaction:

$$2\,NO + O_2 \rightarrow 2\,NO_2 \qquad (21)$$

However, this reaction is a three-body reaction, governed by the following the kinetic equation:

$$dNO_2/dt = k_{20}\,[NO]^2\,[O_2] \qquad (22)$$

Thus, at high concentrations of nitric oxide the reaction is fast. At low concentrations the nitric oxide squared term becomes very small, and the reaction becomes unimportant under atmospheric conditions. In the tailpipe of a vehicle where the nitric oxide concentrations are high, some nitrogen dioxide is produced. Once the tailpipe exhaust is mixed with air, the reaction is quenched because of dilution. Typically, the exhaust gas is 90% or more nitric oxide. This is because exhaust gases are quite hot, and nitrogen dioxide undergoes thermal decomposition to nitric oxide in the exhaust stream.

Nitrogen dioxide is a brown gas that undergoes photolysis in the atmosphere in the wavelength region 290–430 nm, as noted above in section “Photochemistry — Some Basics”:

$$NO_2 + h\nu \rightarrow NO + O \qquad (23)$$

The oxygen atom produced reacts rapidly with molecular oxygen in the troposphere to form ozone:

$$O + O_2 + M \rightarrow O_3 + M \qquad (24)$$

Here M is a third body (typically nitrogen, oxygen, or argon, the most abundant species in air). The thermal reaction with nitric oxide is quite fast:

$$NO + O_3 \rightarrow NO_2 + O_2 \quad (25)$$

Balancing chemical equations 22–25 shows that the formation of ozone depends on the amount of nitrogen dioxide relative to nitric oxide and the intensity of the actinic light that allows the photolysis of the nitrogen dioxide. Indeed, these reactions do not lead to net production of ozone beyond that due to the nitrogen dioxide initially emitted into the air as a primary pollutant.

#### *Free Radicals*

In the Los Angeles air basin and surrounding areas the observations were different. Haagen-Smit showed that the photolysis of hydrocarbons and nitrogen dioxide forms large amounts of ozone[1,2,8]. Free radicals are responsible, as Leighton indicated[13]. However, the mechanism was not understood. Francis Blacet at the University of California, Los Angeles, showed that nitrogen dioxide is the source of the oxygen atom needed to form the ozone[1,2]. However, nitric oxide must be converted to nitrogen dioxide without reaction with ozone to accomplish net production of ozone (as in Haagen-Smit's experiments and as observed in the urban atmosphere). The necessity for nitric oxide conversion, plus the fact that the carbon monoxide released as a primary pollutant was also being removed from the air basin by some chemical or biochemical process, led to the proposal that hydroxyl radicals might be responsible for the oxidation [1,2,43,44,45,46].

### Volatile Organics in Urban Air

#### *Carbon Monoxide and Methane*

In combustion reactions, hydroxyl radical was known to be an important species because it can react with carbon monoxide to produce the hydroperoxyl radical[1,2]. Researchers proposed that ozone photolysis could produce hydroxyl radical via the reaction of excited oxygen atoms with water vapor:

$$O_3 + h\nu \rightarrow O_2 + O\,(^1D) \quad (26)$$

$$O\,(^1D) + H_2O \rightarrow 2\,OH \quad (27)$$

The reaction of hydroxyl radical with carbon monoxide in polluted air would lead to the formation of H atoms and subsequently the hydroperoxyl radical in the presence of molecular oxygen:

$$OH + CO \rightarrow CO_2 + H \quad (28)$$

$$H + O_2 \rightarrow HO_2 \quad (29)$$

Reaction of hydroperoxyl radical with nitric oxide would produce nitrogen dioxide and hydroxyl radical:

$$HO_2 + NO \rightarrow NO_2 + OH \quad (30)$$

The subsequent chain reaction with hydroxyl radical and carbon monoxide would then lead to the chemical formation of nitrogen dioxide and, ultimately, to ozone in sunlight.

This same chemistry could also occur with methane ($CH_4$) if the hydroxyl radical abstracted a hydrogen atom and formed the methyl radical ($CH_3$) and subsequently the methylperoxy radical, which can react with nitric oxide to form nitrogen dioxide:

$$OH + CH_4 \rightarrow CH_3 + H_2O \quad (31)$$

$$CH_3 + O_2 \rightarrow CH_3O_2 \quad (32)$$

$$CH_3O_2 + NO \rightarrow CH_3O + NO_2 \quad (33)$$

The methoxy radical ($CH_3O$) subsequently can react with molecular oxygen to form formaldehyde and hydroperoxyl radical:

$$CH_3O + O_2 \rightarrow HCHO + HO_2 \quad (34)$$

Hydroperoxyl radical can, of course react with nitric oxide, as noted earlier (reaction 30) to form nitrogen dioxide and hydroxyl radical and contribute to the chain reactions leading to net nitrogen dioxide production and subsequently ozone production upon the photolysis of nitrogen dioxide (reactions 8 and 9). Formaldehyde can photolyze to form another hydrogen atom and the formyl radical (HCO):

$$HCHO + h\nu \rightarrow HCO + H \quad (35)$$

This process can be followed by the reaction of both products with oxygen to form carbon monoxide and hydroperoxyl radical. Alternatively, formaldehyde can react with hydroxyl radical by abstraction to form the formyl radical and water:

$$HCHO + OH \rightarrow HCO + H_2O \quad (36)$$

Here the subsequent reaction of the formyl radical with oxygen will lead to the formation of carbon monoxide and hydroperoxyl radical. Thus, both methane and carbon monoxide would react to form hydroperoxyl radical. Hydroperoxyl radical, in turn, could oxidize nitric oxide to nitrogen dioxide (reaction 30), which can subsequently form ozone upon photolysis (reactions 8 and 9).

### *Other Volatile Organics*

The chemical reactivity and concentrations of methane and carbon monoxide in urban atmospheres were not sufficient to explain the observed ozone production in urban air. Researchers quickly realized that many other volatile organics being emitted into the air from incomplete combustion and industrial processes were contributing to the observed formation of ozone and other oxidants. These organic compounds included alkanes, alkenes, and aromatic emissions that could react with hydroxyl radical to produce peroxy radicals in the presence of oxygen.

The hydroxyl radical reactions proceed via addition or abstraction. Addition reactions with hydroxyl radical occur when the reactive hydrocarbons have sites of unsaturation (double bonds), as in the reactive olefins or aromatic hydrocarbons. Abstraction reactions occur in alkanes or aldehydes, where the hydrogen bonds are broken on the basis of their bond strengths and numbers.

Thus, the formation of high ozone levels in urban centers involves the photochemical production of ozone from volatile hydrocarbons. These hydrocarbon emissions serve as the fuel for chain reactions leading to the oxidation of nitric oxide to nitrogen dioxide in the troposphere. Volatile hydrocarbons are released into the air during incomplete combustion of fuels. Sources include gasoline and diesel engines; power plants; chemical industries; and evaporative losses from gas stations, vehicles, etc. Another source is vegetative emissions. Indeed, on a global scale the natural release of volatile hydrocarbons from vegetation (on a mass basis) exceeds worldwide anthropogenic releases[47].

The relative reactivity of hydrocarbons with hydroxyl radical varies with the chemical structure of the hydrocarbon. Some relative rate constants for hydroxyl radical reactions with different classes of hydrocarbons are given in Table 1.

**TABLE 1**
**Gas-Phase Hydroxyl Radical Reactivities of Some Important Volatile Organic Compounds**

| Compound | Rate of Reaction with Hydroxyl Radical ($cm^3$ $molecule^{-1}$ $s^{-1}$) | Relative Rate |
|---|---|---|
| Methane | $6.86 \times 10^{-15}$ | 1.0 |
| Ethane | $2.57 \times 10^{-13}$ | 37.5 |
| Propane | $1.15 \times 10^{-12}$ | 167.6 |
| *n*-Butane | $2.54 \times 10^{-12}$ | 370.3 |
| 2-Methylpropane | $2.33 \times 10^{-12}$ | 339.7 |
| *n*-Pentane | $3.96 \times 10^{-12}$ | 577.3 |
| 2,2-Dimethylpropane | $8.49 \times 10^{-13}$ | 123.8 |
| 2,3-Dimethlybutane | $5.99 \times 10^{-12}$ | 873.2 |
| Cyclopropane | $8.4 \times 10^{-14}$ | 12.4 |
| Cyclobutane | $1.5 \times 10^{-12}$ | 218.7 |
| Cyclopentane | $5.08 \times 10^{-12}$ | 740.5 |
| Cyclohexane | $7.49 \times 10^{-12}$ | 1091.8 |
| Ethene | $8.52 \times 10^{-12}$ | 1242.0 |
| Propene | $2.63 \times 10^{-11}$ | 3833.8 |
| 1-Butene | $3.14 \times 10^{-11}$ | 4577.3 |
| Cyclohexene | $6.77 \times 10^{-11}$ | 9868.8 |
| 1,3-Butadiene | $6.66 \times 10^{-11}$ | 9708.5 |
| Isoprene | $1.01 \times 10^{-10}$ | 14723 |
| Limonene | $1.71 \times 10^{-10}$ | 24927 |
| α-Pinene | $5.37 \times 10^{-11}$ | 7828 |
| β-Pinene | $7.89 \times 10^{-11}$ | 11,501.5 |
| Terpinolene | $2.25 \times 10^{-10}$ | 32,798.8 |
| Formaldehyde | $9.2 \times 10^{-12}$ | 1341.1 |
| Acetaldehyde | $1.6 \times 10^{-11}$ | 2332.4 |
| Methanol | $9.3 \times 10^{-13}$ | 135.6 |
| Ethanol | $3.3 \times 10^{-12}$ | 481.0 |
| Peroxyacetyl nitrate | $1.1 \times 10^{-13}$ | 16.0 |

Data are for the room-temperature reaction at 298 K and at 1 atm pressure. Relative rates are compared to that for methane. Data are from the recent evaluation by Atkinson[42].

### Alkanes

The reactions of alkanes with hydroxyl radical proceed by abstraction and lead to the formation of alkoxy radicals. The numbers and types of hydrogens available for abstraction and their relative bond strengths determine an alkane's reactivity. Thus, for normal and branched alkanes, the order of reactivity is tertiary > secondary > primary. The abstraction reactions can be estimated fairly accurately by making assumptions about the relative reactivity of the various types of hydrogens on the compounds and their numbers on the molecules[48]. This procedure allows the initial rates of oxidation and attack to be estimated accurately for many compounds from experimentally determined rate constants and product analysis studies[1,2].

### Unsaturated Organics

For unsaturated organics such as alkenes (olefins) and aromatic compounds, hydroxyl radical can react by addition to the compounds to form hydroxy radical compounds. These can, in turn, combine with oxygen to form hydroxyperoxy radical species. The simplest case (for ethene [$H_2C=CH_2$]) is as follows:

$$\begin{array}{l} \qquad\qquad\qquad\qquad\quad H\backslash \\ H_2C=CH_2 + OH \rightarrow H\text{-}C - C\text{-}H \\ \qquad\qquad\qquad\qquad HO/ \quad \backslash H \end{array} \qquad (37)$$

$$\begin{array}{l} \quad H\backslash \qquad\qquad\qquad\quad H\backslash \quad\; /O\text{-}O \\ \quad H\text{-}C - C\text{-}H + O_2 \rightarrow H\text{-}C - C\text{-}H \\ HO/ \quad \backslash H \qquad\qquad HO/ \quad \backslash H \end{array} \qquad (38)$$

The peroxy radical formed in reaction 38 can react with nitric oxide to form the 2-hydroxy substituted ethoxy radical, $CH_2OH\text{-}HCHO$, which either can undergo decomposition to form formaldehyde and the methylene hydroxy radical, $CH_2OH$, or can react with molecular oxygen to form glycoaldehyde ($CH_2OH\text{-}CHO$) and the hydroperoxyl radical. For the reaction of ethene with hydroxyl radical and the subsequent reaction with nitric oxide, the decomposition to form formaldehyde and $CH_2OH$ is the primary pathway. Similarly, larger olefins primarily undergo similar reactions[1,2]. In the presence of high nitric oxide levels, the peroxy radicals formed can also react to a small extent (10%) to form hydroxy nitrates[1,2,49].

Internal olefins such as trans-2-butene ($CH_3CH=CHCH_3$) react with hydroxyl radical in the presence of oxygen to form the 2-hydroxy-3-peroxy radical. In the presence of nitric oxide this pathway can account for about 10% of the hydroxy nitrate product and acetaldehyde, as follows:

$$\begin{array}{l} \qquad\qquad\qquad\qquad\qquad\quad HO\backslash \qquad \cdot \\ OH + CH_3CH=CHCH_3 \rightarrow CH_3\;CH - CHCH_3 \end{array} \qquad (39)$$

$$\begin{array}{l} HO\backslash \qquad \cdot \qquad\qquad\qquad HO\backslash \quad /OO\cdot \\ \;CH_3CH - CHCH_3 + O_2 \rightarrow CH_3CH - CHCH_3 \end{array} \qquad (40)$$

$$\begin{array}{l} HO\backslash \quad /OO\cdot \qquad\qquad\qquad HO\backslash \quad /ONO_2 \\ \;CH_3CH - CHCH_3 + NO \rightarrow CH_3CH - CHCH_3 \end{array} \qquad (41a)$$

$$\begin{array}{l} HO\backslash \quad /OO\cdot \qquad\qquad\qquad HO\backslash \quad /O\cdot \\ \;CH_3CH - CHCH_3 + NO \rightarrow CH_3CH\text{-}\;CHCH_3 + NO_2 \end{array} \qquad (41b)$$

$$\overset{HO}{CH_3CH}-\overset{O\cdot}{CHCH_3} \rightarrow CH_3CHOH + CH_3CHO \qquad (42)$$

$$CH_3CHOH + O_2 \rightarrow CH_3CHO + HO_2 \qquad (43)$$

Thus, the reaction of hydroxyl radicals with unsaturated alkenes can lead to the direct formation of aldehydes and organohydroxy nitrates in the presence of nitric oxide and oxygen[1,2,49].

Note that the reaction of olefins can lead to the formation of hydroxy-substituted compounds. Similarly, the reactions of large primary alkoxy radicals formed by the abstraction reaction of hydroxyl radical with a large alkane can also result in the formation of these types of products via 1,4- and 1,5-hydrogen shift reactions[1,2]. The 1,5-hydrogen shifts are favored kinetically, as they proceed via a six-membered cyclic transition state. The 1,4-hydrogen shift reactions are slower because of the more sterically hindered five-member cyclic transition state. Schematically, the reaction for *n*-pentoxyl radical is as follows:

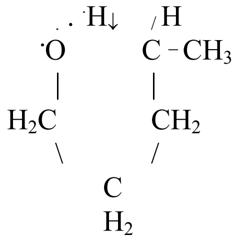

$$\rightarrow HOCH_2CH_2CH_2\dot{C}HCH_3 \qquad (44)$$

The radical formed can combine with oxygen to yield the isomerized hydroxyperoxy radical species. Such isomerizations are quite rapid. The isomerization reaction competes effectively with hydrogen abstraction by molecular oxygen to form the more conventional aldehyde product; in many cases this is the major pathway for reaction. These pathways and alkoxy radicals subsequently formed by reaction of the hydroxyperoxy radicals with nitric oxide yield hydroxyaldehyde products that can be important components of the semivolatile organics associated with secondary organic aerosols.

The large alkyl peroxy radical species can undergo isomerization when they react with nitric oxide to form the organonitrate addition products[1,2,49]. Thus, the reaction of the larger peroxy radicals with nitric oxide can lead to significant formation of alkyl nitrates as products instead of nitrogen dioxide and alkoxy radical (RO). As Atkinson and Lloyd noted[49], for the larger alkyl groups these products can account for appreciable amounts of the reaction pathway and can become very important in modeling ozone formation, as they constitute an alternative sink for the nitrogen oxides that are important for the ozone-forming chain reactions with various hydrocarbons. The reader is referred to the recent textbooks on this subject for further discussion of the free-radical reactions and isomerization chemistries[1,2].

### Aromatic Hydrocarbons

Aromatic hydrocarbons can also react with hydroxyl radical. The addition reaction has been observed to form ring-opened products[1,2], consistent with mechanistic studies on toluene and ground-state oxygen atoms, which are also strongly electrophilic[50]. The addition reactions in the presence of nitrogen dioxide lead to the formation of nitrophenolic products that are quite water soluble and are also phytotoxic. This is an area where mechanistic studies are still needed. Essentially, the ring-opened products react faster by many orders of magnitude than the starting aromatic compounds, thus making it very difficult to conduct product studies that are not complicated by significant yields of secondary products.

### Interactions and Feedbacks of Natural Hydrocarbons

Not only do the anthropogenic hydrocarbons undergo free-radical oxidation reactions with hydroxyl radical, but naturally occurring hydrocarbon emissions from plants and trees do the same. Of particular importance are the volatile hydrocarbons from deciduous and coniferous forests. Deciduous trees (hardwoods) are typically very significant emitters of isoprene. This hemiterpene and its dimers, the monoterpenes, have been known as important species in the chemistry of the troposphere for some time [e.g., 47,51].

The natural hydrocarbons are very reactive. They have relatively short lifetimes in the atmosphere, as they will react with hydroxyl radical, ozone, and nitrate radical. Isoprene oxidations lead to the formation of formaldehyde, methylvinyl ketone, and methacrolein as primary oxidation products[52]. Indeed, the production of formaldehyde from isoprene emissions in deciduous forests will lead to the formation of hydrogen peroxide in areas where the nitric oxide levels are low. Thus, isoprene (2-methyl-1,3-butadiene) oxidation will yield formaldehyde and other aldehyde products. These aldehyde products will lead to the further formation of hydroperoxyl radical by photolysis and reactions with hydroxyl radical. Hydroperoxyl radical can react with itself to form hydrogen peroxide and oxygen if nitric oxide is at low levels. Hydrogen peroxide can be an important oxidant for sulfur dioxide conversion to secondary sulfate, as we discuss below in the section “Acid Rain and Its Connections to Oxidant Chemistry”.

#### *Monoterpenes*

A number of the monoterpenes can react with ozone at rates that are as fast as or faster than the rates of their reactions with hydroxyl radical. Such production of hydroxyl radical has been observed by a number of researchers in the laboratory[53,54,55]. The initial adduct is a primary ozonide (molozonide intermediate)[1,2]. The energetic Criegee intermediates that are formed when the ozone-bridged olefinic bond breaks can lead to a wide number of products including bi-radicals and hydroperoxides[2], as well as the hydroxyl radical[53,54,55].

The production of hydroxyl radical by the thermal reaction of ozone with alkenes, in this case monoterpenes, might be an important nighttime source of hydroxyl radical[2,53,54,55]. Because the reaction rates for ozone with monoterpene hydrocarbons are quite fast, one can calculate that the more reactive monoterpenes can lead directly to a significant source of hydroxyl radical in coniferous forests without the need for photochemistry. This source can account for hydroxyl radical observed at nighttime in forested areas.

#### *Isoprene*

Isoprene is the most abundant of the natural hydrocarbon emissions[47,51]. As we continue to control emissions of volatile reactive hydrocarbons from combustion and other sources, isoprene and its monoterpene cousins are playing an increasingly important role in regional and even urban ozone formation. This is due to the increasing levels of nitrogen oxide emissions and the use of catalytic converters to reduce volatile hydrocarbon emissions. Thus, ozone formation by chain reactions involving nitrogen oxides and volatile hydrocarbons is enhanced by the interactions of the natural hydrocarbons with primary anthropogenic nitrogen oxide emissions.

### *Summing Up the Atmospheric Chemistry of Volatile Organics*

To summarize, photochemical oxidation of volatile hydrocarbons, natural and anthropogenic, is dominated by hydroxyl radical reactions. These reactions lead to the formation of peroxy radicals or hydroxyperoxy radicals via initial abstraction and addition reactions. The subsequent reactions of these radicals with molecular oxygen lead to the formation of aldehydes and the production of hydroperoxyl radical. The peroxy radicals can react with nitric oxide to form nitrogen dioxide, which, in turn, can photolyze to yield oxygen atoms that result in the enhancement of tropospheric ozone levels.

### Aldehyde Oxidation and the Formation of Peroxyacyl Nitrates

The oxidation of aldehydes by hydroxyl radical proceeds by abstraction; for example, acetaldehyde reacts with hydroxyl radical and then oxygen addition to form the peroxyacetyl radical ($CH_3CO\text{-}OO$):

$$OH + CH_3CHO \rightarrow CH_3CO + H_2O \quad (45)$$

$$CH_3CO + O_2 \rightarrow CH_3CO\text{-}OO \quad (46)$$

The peroxyacetyl radical can react with nitric oxide to form a number of nitrogen dioxide molecules, carbon dioxide, formaldehyde, and hydroxyl radical via the following reaction scheme:

$$CH_3CO\text{-}OO + NO \rightarrow CH_3COO + NO_2 \quad (47)$$

$$CH_3COO \rightarrow CH_3 + CO_2 \quad (48)$$

$$CH_3 + O_2 \rightarrow CH_3O_2 \quad (49)$$

$$CH_3O_2 + NO \rightarrow CH_3O + NO_2 \quad (50)$$

$$CH_3O + O_2 \rightarrow HCHO + HO_2 \quad (51)$$

$$HO_2 + NO \rightarrow NO_2 + OH \quad (52)$$

The peroxyacetyl radical can also react with nitrogen dioxide to form a reasonably stable compound with which it is in thermal equilibrium — peroxyacetyl nitrate:

$$CH_3CO\text{-}OO + NO_2 \leftrightarrow CH_3CO\text{-}OO\text{-}NO_2 \quad (53)$$

Many of the higher aldehydes will form peroxyacyl nitrates. These compounds were first discovered in the early 1950s and now are known to play important roles in the tropospheric chemistry of urban, regional, and global atmospheres, primarily because they are relatively long lived. Their reactivity with hydroxyl radical is low (Table 1), and they have a slow photolysis rate that is probably important only at altitudes above 7 km[2]. The solubilities of the peroxyacyl nitrates in water are low, making wet removal and hydrolysis under the acidic conditions found in most aerosols and cloud droplets slow, as well. Removal occurs mainly by thermal decompositions via reaction 53, with subsequent loss of the peroxyacetyl radical by reaction with nitric oxide, as noted in reactions 47–52, or by reaction with hydroperoxyl radical in areas with low levels of nitric oxide to form peracetic acid ($CH_3CO\text{-}OOH$):

$$CH_3CO\text{-}OO + HO_2 \rightarrow CH_3CO\text{-}OOH + O_2 \tag{54}$$

The peroxyacyl nitrates are important air pollutant species. The following section considers the history and properties of this key class of oxidants and measurement methods for them.

### Synthesis, Properties, and Measurement of Peroxyacyl Nitrates

#### *Early Synthesis and Isolation*

The first routine method for synthesis and isolation of peroxyacetyl nitrate was developed by Edgar Stephens and colleagues at UCR[17]. Ethyl nitrite ($CH_3CH_2ONO$) was photolyzed in air to produce the compound. This chemistry is represented in the following processes:

$$CH_3CH_2ONO + h\nu \rightarrow CH_3CH_2O + NO \tag{55}$$

$$CH_3CH_2O + O_2 \rightarrow HO_2 + CH_3CHO \tag{56}$$

$$HO_2 + NO \rightarrow NO_2 + OH \tag{57}$$

$$OH + CH_3CHO \rightarrow CH_3CO + H_2O \tag{58}$$

$$CH_3CO + O_2 + NO_2 \rightarrow CH_3CO\text{-}OO\text{-}NO_2 \tag{59}$$

The reaction products were gas chromatographed on preparatory-sized columns to separate and collect the peroxyacetyl nitrate with cryogenic trapping. The compound was placed in large air canisters, diluted in zero air, and stored in a cold room for use in fumigation and chemistry studies. This approach was quite successful, and a number of publications on the toxicity of peroxyacetyl nitrate and its chemical and physical properties resulted[56,57].

Laboratory accidents reported with use of this photoreactor system[56] were attributed to the condensation of the explosive peroxyacyl nitrates in a vacuum or to pressure gauge systems that caused small explosions and destruction of the vacuum gauges. Other photochemical production methods employed followed the general photochemical reaction scheme for halogens, where X is chlorine or bromine:

$$X_2 + h\nu \rightarrow 2\,X \tag{60}$$

$$X + CH_3CHO \rightarrow CH_3CO + HX \tag{61}$$

$$CH_3CO + O_2 + NO_2 \rightarrow CH_3CO\text{-}OO\text{-}NO_2 \tag{62}$$

Similarly, nitrate radical generated by the dark reaction of nitrogen dioxide and ozone has been used to produce peroxyacetyl nitrate (reaction 11)[56].

These methods require photochemical apparatus and chromatographic or distillation equipment to separate the peroxyacetyl nitrate for instrument calibration or laboratory study. The fact that the compound can considered a mixed anhydride of peracetic acid and nitric acid[57] led Hendry and co-workers to use the strong acid nitration of peroxyacetic acid in aqueous solution to synthesize peroxyacetyl nitrate, followed by extraction into a *n*-alkane solvent[56]. Gaffney and co-workers modified the procedure to overcome potential safety hazards of working with peroxyacetyl nitrate dissolved in a highly volatile solvent and recommended that *n*-tridecane be used for the synthesis of high-purity peroxyacetyl nitrate and derivatives[58].

### Improved Analysis and Characterization

Over the years, advances in the synthesis of peroxyacyl nitrates have led to improvements in analytical procedures for them and determinations of their chemical and physical properties. Noteworthy are studies of Karen Darnall and James N. Pitts, Jr., who used canister samples obtained from Edgar Stephens to produce high-concentration peroxyacetyl nitrate samples in deuterated chloroform and methylene chloride. These studies led to the understanding that peroxyacetyl nitrate is highly soluble in nonpolar solvents. The researchers used nuclear magnetic resonance (NMR) to study the liquid-phase reactions of this novel oxidant with olefins; studies examining alcohols and related compounds followed[56,57].

Key structural information was obtained by Stephens and by Nicksic and colleagues, who observed products of base hydrolysis that helped to confirm the structure of peroxyacetyl nitrate[59]. Later studies with ammonia and amines that showed the formation of oxygen, nitrous acid, and acetamides supported these observations[60].

The very simple ultraviolet absorption spectrum of peroxyacetyl nitrate observed by Stephens was confirmed by Senum, Lee, and Gaffney at Brookhaven National Laboratory in the early 1980s[56,57]. The absorption spectrum shows essentially no significant features or band structure and minimal absorption above 290 nm. Recent reexamination of the data led to the conclusion that the peroxyacetyl nitrate molecule is not readily photolyzed at altitudes below 5–7 km. In 1984, Tim Wallington, Roger Atkinson, and Arthur Winer at UCR showed the reaction rates with hydroxyl radical to be quite slow. The aqueous-phase solubilities of peroxyacetyl nitrate were examined by Yin-Nan Lee of Brookhaven and found to be 3–5 M $atm^{-1}$; these results were confirmed by Chester Spicer's group at Battelle Columbus Laboratory[56,57].

### Thermal Decomposition

In the meantime, peroxyacetyl nitrate was observed by Stephens and others to decompose thermally to form methyl nitrate, carbon dioxide, and other minor products. What has been determined is that peroxyacetyl nitrate is in thermal equilibrium with nitrogen dioxide and the peroxyacetyl radical (reaction 53) and that this equilibrium is strongly temperature dependent[1,2,56,57]. At the cold temperatures found at higher altitudes and in wintertime, peroxyacetyl nitrate is quite stable and has a lifetime approaching months. At lower altitudes and at higher temperatures (summer), peroxyacetyl nitrate has a fairly short lifetime that depends on the ratio of nitric oxide to nitrogen dioxide and the abundance of hydroperoxyl radical, which can remove the peroxyacetyl radical and form peroxyacetic acid. Thus, at high nitric oxide concentrations the peroxyacetyl radical reacts, and peroxyacetyl nitrate is not reformed. At very low nitric oxide concentrations, hydroperoxyl radical levels can be elevated, and this species can react with the peroxyacetyl radical to remove peroxyacetyl nitrate.

Hanwant Singh was the first to point out that peroxyacetyl nitrate can act as a means of transporting nitrogen dioxide over very long distances and therefore is a globally important molecule, not just an urban air pollutant[61].

### Early Measurements

The first measurements of peroxyacetyl nitrate were made in the laboratory by using long-path infrared techniques that worked reasonably well at the higher concentrations found in the Los Angeles air shed[62,63]. However, the infrared instruments of the early 1960s were clearly incapable of routine monitoring of peroxyacetyl nitrate. With the invention of the electron capture detector (ECD) by James Lovelock, Ellis Darley and co-workers in 1963 developed a method for analysis that has been used since quite successfully[16]. Many others have adopted a similar approach that employs a nonpolar column to separate peroxyacyl nitrates from other ECD-sensitive gases (oxygen, Freons, organic nitrates, etc.)[57]. Used in the 1960s and 1970s to monitor peroxyacetyl nitrate in the air in the Riverside area, this method found very high values of the phytotoxin (20–50 ppb). In 1974, James Lovelock and Stewart Penkett used a similar analysis system to measure peroxyacetyl nitrate off the coast of England, observing a level of 2 ppb and alerting the community to the compound's ubiquity[64].

Calibration of the early instruments was usually accomplished by using base hydrolysis to yield acetate and nitrite. These methods had some difficulties, and thermal decomposition to nitrogen dioxide and direct measurement with a chemiluminescent nitrogen oxides monitor have seen more use in recent years[57].

### Improved Measurements

Other approaches were examined in the mid-1980s, when luminol chemiluminescence was observed to occur with peroxyacetyl nitrate, as well as with nitrogen dioxide. Two groups, Gaffney's at Los Alamos and Stedman's at the University of Denver, demonstrated in 1986 that chromatography works for this application. Stedman published the method in 1988, documenting a 5-min analysis for peroxyacetyl nitrate with a packed column[65]. Gaffney and Marley reported a modification of this method that employs fast capillary chromatography to obtain 30-s time resolution for peroxyacetyl nitrate and its analogs and also gives measurements of nitrogen dioxide[66].

Mass spectrometry of peroxyacetyl nitrate in electron impact mode was first reported by Ron Steer, Karen Darnall, and James N. Pitts, Jr., in 1969. The chemical ionization spectrum was reported in 1976 by Chris Pate, Jerry Sprung, and Pitts, who used one of the first Finnigan gas chromatograph-mass spectrometer systems equipped with this capability. Because of its very large ECD cross-section, the negative-ion mass spectrometric sensitivity for peroxyacetyl nitrate is quite high. This was predicted in 1989 to be a reasonable approach[56], and it has been pursued in recent years by Chester Spicer of Battelle Columbus and by Hiroshi Tanimoto and Hajime Akimoto in Japan[67,68].

Because of the compound’s rather undistinctive ultraviolet spectrum, direct ultraviolet absorption spectroscopy has not been used for peroxyacetyl nitrate analyses. Instead, NMR has been used for laboratory studies, and that method, along with circular internal reflectance infrared spectroscopy, may be important in future studies of peroxyacyl nitrates in solution.

### The Worldwide Family of Peroxyacyl Nitrates

Since the initial work at the Franklin Institute in Philadelphia and the original observation of peroxyacyl nitrates in Los Angeles photochemical smog, these compounds have been measured in every corner of the world[56,57]. Measured peroxyacyl nitrates include the most abundant, peroxyacetyl nitrate, as well as peroxypropionyl nitrate and peroxy-*n*-butyryl nitrate. Other peroxyacyl nitrates deemed important include those derived from isoprene oxidation (MPAN)

and aromatics (PBzN)[56,57]. Recently, peroxyisobutyryl nitrate (PiBN) has been reported by Roberts and co-workers at the Symposium on Atmospheric Chemistry at the National American Meteorology Society Meeting in Orlando, Florida in January 2002 (work in press). See Table 2 for structures.

**TABLE 2**
**Chemical Structures of Peroxyacyl Nitrates (Peracylnitric Anhydrides)**

| Structure | Name | Abbreviation |
|---|---|---|
| $CH_3CO$-OO-$NO_2$ | Peroxyacetyl nitrate | PAN |
| $CH_3CH_2$-CO-OO-$NO_2$ | Peroxypropionyl nitrate | PPN |
| $CH_3CH_2CH_2CO$-OO-$NO_2$ | Peroxy-*n*-butyryl nitrate | PnBN |
| $(CH_3)_2CH$-CO-OO-$NO_2$ | Peroxy-*i*-butyryl nitrate | PiBN |
| $CH_2=C(CH_3)$ -CO-OO-$NO_2$ | Peroxymethacryloyl nitrate | MPAN |
| $C_6H_5$-CO-OO-$NO_2$ | Peroxybenzoyl nitrate | PBzN |

### *Peracids*

The peroxyacyl nitrates are strongly connected with peracids, as noted earlier (see reaction 54) by the reaction of the peroxyacyl radicals with hydroperoxyl radical at low concentrations of nitric oxide and nitrogen dioxide. The peracids are known to be produced in rural air masses, where nitric oxide levels allow the buildup of hydroperoxyl radical. Therefore, methods for measurement of these species and other organic peroxides need to be developed if their concentrations and impacts are to be assessed. This will especially be the case as we begin to control $NO_x$ emission levels to decrease urban and regional ozone levels.

### *Recent Drops in Peroxyacetyl Nitrate Levels*

In recent years, peroxyacetyl nitrate levels have dropped significantly in urban U.S. regions like the Los Angeles air basin and surrounding air shed. Levels above 5 ppb are rarely observed, a tenfold decrease since the 1960s and 1970s. However, in other areas of the world, peroxyacyl nitrates can also be produced in large quantities if $NO_x$ levels and the reactive hydrocarbon species leading to peroxyacetyl nitrate and ozone formation are not controlled. Indeed, in Mexico City we recently measured total peroxyacyl nitrates at 40 ppb by using gas chromatography with ECD[69].

### *Alternative Fuels and Peroxyacyl Nitrates*

The use of alternative oxyfuels might enhance production of peroxyacyl nitrates, as these fuels are known to enhance aldehyde emissions. This topic will be discussed further in the section “Alternative Fuels”, below. Oxygenated fuels that lead to increased emissions of larger aldehydes or aldehyde precursors such as ethanol or methyl-*t*-butyl ether (MTBE) will require assessment of the environmental impacts of peroxyacyl nitrates and other oxidants[70,71,72], not simply ozone or carbon monoxide reductions. In addition, the potential for long-range transport of peroxyacyl nitrates on continental scales and for the compounds to function as a source of regional ozone should not be overlooked in assessments of fuel options[56,57,61].

### Other Organic Nitrates

The peroxyacyl nitrates are indicators of both peroxy radical activity and the potential importance of other organic nitrates. The decomposition of peroxyacetyl nitrate is known to lead to the formation of methyl nitrate, which is fairly stable in the atmosphere[73,74]. The nighttime thermal decomposition of methyl nitrate might be an important source of radicals for the oxidation of nitric oxide to nitrogen dioxide and for the formation of nitrous acid. Better understanding of the chemical toxicity and radiative behavior of these compounds is needed. A number of research groups, notably Paul Shepson's at Purdue University[57], are beginning to explore the organic nitrates in detail by using methods mentioned above.

Peroxy nitrates might play important roles in the transport of nitrogen dioxide under cold conditions. These compounds are formed by the combination reaction of nitrogen dioxide with alkyl peroxy radicals, which is similar to the peroxyacetyl nitrate equilibrium:

$$RO_2 + NO_2 \leftrightarrow RO_2NO_2 \tag{63}$$

These species were first examined by Edward Edney, John Spence, and Philip Hanst at the U.S. Environmental Protection Agency laboratories. Because of their short lifetimes at room temperature, the compounds were assumed not to have a key storage role in the lower atmosphere[75]. However, at middle latitudes in the wintertime they may have lifetimes that approach days, and, like peroxyacetyl nitrate (depending on nitric oxide, hydroperoxyl radical, and nitrogen dioxide levels), they might be reformed to actively transport nitrogen dioxide and also tie up peroxy radicals that would lead to ozone formation.

### Production of Aromatic Compounds

In laboratory studies, the peroxyacyl nitrates are known to be quite sensitive to walls; therefore, they are likely to react on aerosol surfaces. These compounds are very soluble in nonpolar organics and indeed may undergo important oxidation reactions on soot surfaces, leading to the formation of oxidized and nitrated polynuclear aromatic hydrocarbons potentially having high mutagenicity[2].

### Summing Up Peroxyacyl Nitrates

We have known about peroxyacyl nitrates for over 40 years, but our fundamental understanding of and ability to measure these fascinating molecules are just now beginning to expand. Interestingly, many physical chemists are beginning to explore the unusual properties of these highly energetic molecules. The area of peroxyacetyl nitrate chemistry, opened to us by Edgar Stephens and his colleagues, looks bright, and its further exploration should lead us to a much better understanding of the key oxidation reactions occurring in the troposphere.

### Primary and Secondary Aerosols

Besides the gaseous air pollutants, a number of atmospheric species exist in particulate or aerosol phase. Aerosols are solid or liquid particles that are suspended in the air and are in equilibrium with the gaseous atmosphere around them. Water vapor is, of course, a major component of tropospheric gases. Liquid water in the form of cloud droplets or aerosols is also an important species that can incorporate gaseous pollutants, especially water-soluble ones. Particulate material

(aerosols) can be pure compounds or complex mixtures, hydroscopic or hydrophobic. Aerosols can transport a wide variety of inorganic and organic pollutants significant distances if the sizes are in the submicron range.

### Primary Aerosols

Primary aerosols are emitted directly into the atmosphere in particulate or aerosol form. An example of a primary aerosol is the carbonaceous soot formed during incomplete combustion processes. Diesel soot is a classic example of this form of primary carbonaceous aerosol. Another example of primary aerosols would be fly ash from coal burning or incineration of wastes. This material is typically generated by high-temperature processes that result in the production of condensed inorganic materials formed as small spherical beads, typically high in silicates or iron oxides. Sources of natural primary aerosols include sea salt spray, which forms hydrated sodium chloride particles and mixed salt aerosols; volcanic ash emissions; natural fires producing soots; and wind-blown dusts. Anthropogenic sources of primary aerosol and particulate material include mechanical abrasion that produces construction and industrial dusts, as well as abrasion of tires and pavement materials on roads[1,2,76].

Primary aerosols produced by combustion sources are usually much smaller in size than aerosols from mechanical or wind-driven sources[1,2,76]. Combustion-derived aerosols are usually carbonaceous soots from mobile diesel and internal combustion engine sources and inorganic fly ash from coal-fired stationary power sources. Diesel soot particles emitted into the atmosphere from exhaust systems are typically 0.1–0.6 μm in diameter. In the absence of external controls, fly ash particles are typically larger, 1–5 μm in diameter. Considerable improvement in the removal of particulates from power plant plumes has significantly reduced the airborne levels of the coal-derived primary aerosols. However, carbonaceous soots that have not been controlled make up a significant portion of the carbonaceous aerosols found in the troposphere, and they are a major constituent of urban aerosols. This primary soot has been referred to as elemental carbon and black carbon[1,2]. Recent studies in Mexico City found that carbonaceous aerosol loadings (including up to 50% black carbon) accounted for approximately 25% of the fine aerosol, results typical of compositions found in many urban centers[77]. Black carbon is important as a radiative species on urban, regional, and global scales; it is discussed further in the section “Global Scale Impacts”, below.

### Secondary Aerosols

Secondary aerosols are produced by chemical oxidation reactions in the troposphere that yield acidic species or oxidized organics having reduced vapor pressures and increased water solubilities. The end result is condensation of the reaction products on existing particles or dissolution in wet aerosols. The most important aerosol-generating inorganic gases that are released into the atmosphere by human combustion of fossil fuels are the nitrogen oxides (nitric oxide and nitrogen dioxide) and sulfur dioxide. Nitric oxide is oxidized to nitrogen dioxide and subsequently to nitric acid by the reaction of hydroxyl radical with nitrogen dioxide[1,2]. The nitric acid can in turn react with ammonia to form ammonium nitrate, a white solid. Sulfur dioxide in the atmosphere reacts with hydroxyl radical in the gas phase and with hydrogen peroxide and ozone in the aqueous phase to form sulfuric acid. Sulfuric acid is quite water soluble and also has a very low vapor pressure, so it rapidly forms aerosol once it is formed. Sulfuric acid can also react with ammonia to form ammonium bisulfate and ammonium sulfate. These species are all fairly water soluble, and at high relative humidity values they will grow by adding water vapor to their surfaces[1,2]. Sulfate and nitrate make up a substantial amount of the aerosol mass, particularly in the particulate matter less than 2.5 μm in size (PM-2.5).

A natural source of sulfate aerosol is the emission of dimethlysulfide (DMS) from phytoplankton[1,2]. The oxidation of the DMS by hydroxyl radicals yields a series of species that continue to be oxidized, ultimately producing sulfate aerosols. This source of aerosol sulfate is significant globally, but it is typically not responsible for the high sulfate levels seen over the U.S., particularly in the eastern portion of the country. Stationary power plants burning oil and coal are the major source of sulfur dioxide emissions that lead to the formation of aerosol sulfate and are a major source of acidity in the aerosols. Nitrogen oxides (nitric oxide and nitrogen dioxide) are also produced by power plants during combustion, but the most significant sources of these secondary aerosol precursors are the internal combustion and diesel engines in automobiles and trucks[1,2]. Lightning strikes also produce nitric oxide and are an important natural source of this species in lower latitudes where lightning strikes are abundant.

Secondary organic aerosols can be produced by the oxidation of larger organic molecules. In particular, reactions of ozone with olefins can lead to the formation of ozonides and subsequent products having reduced vapor pressures. Natural monoterpene hydrocarbons such as α- and β-pinene are known to produce aerosol when they are oxidized by hydroxyl radical or ozone. These natural monoterpenes can play an important role in the secondary formation of organic aerosols, usually by coating existing particles. However, in clean air they have been credited with the observed formation of fine aerosol due to homogeneous nucleation of oxidized products in forested environments. The isoprene associated with monoterpene emissions from trees and other plants does not lead to significant formation of "heavy" organic products that generate aerosol products. However, as we will see in the next section, isoprene can play an important role in the formation of hydrogen peroxide, which can lead to the oxidation of sulfur dioxide and the formation of regional acidic aerosols and ultimately acid rain.

### Aerosols and Visibility

Aerosols play an important role in the reduction of visibility. Submicron aerosols are very important light-scattering agents. Carbonaceous soots (black carbon) can also absorb light and reduce visibility in urban environments. We will discuss that aspect of the aerosols in the section “Global-Scale Impacts”, below. However, we note here that visibility reduction is of major concern, particularly in the remote areas of the western U.S. where degradation of the national parks is a problem.

### Aerosols and Health

Fine aerosols are known to cause respiratory problems and are currently of interest because of their potential health impacts. Health issues that are correlated with high levels of particulate mass in the PM-10 and PM-2.5 ranges include increases in premature death, respiratory-related hospital admissions, emergency room visits, and observed cases of aggravated asthma and chronic bronchitis. Exposure to fine particles often leads to acute respiratory symptoms, including aggravated coughing, difficult or painful breathing, and decreased lung function potentially experienced as shortness of breath. Along with elevated levels of ozone and other oxidants, particulate air pollution increases pulmonary problems in the population, resulting in increases in work and school absences that lead to decreased productivity.

### Alternative Fuels

Considerable attention has been given to the use of reformulated gasolines and oxygenated fuels — such as methanol-, ethanol-, and MTBE-gasoline blends — as alternative fuels[1,2,78]. The various alternative fuels and their potential air pollution contributions have been reviewed[78]. Oxygenated fuels (2% oxygen content) have been mandated for areas where carbon monoxide levels have been out of compliance, including western metropolitan areas such as Denver, Phoenix, and Albuquerque. We briefly review some issues related to oxygenated fuels, because their use affects oxidant formation chemistry in urban and regional areas. The reader is referred to the review chapter for details on other alternative fuels and their comparative emissions[78].

#### *Aldehyde Formation*

Oxygenated fuels tend to crack during combustion, with formation of aldehydes. The use of methanol or methanol-gasoline blends increases formaldehyde emissions. Ethanol usage increases acetaldehyde emissions, and MTBE usage leads to the emission of isobutene and formaldehyde[78]. As noted in the section on peroxyacetyl nitrate formation, acetaldehyde results in the formation of peroxyacetyl nitrate in urban situations. Indeed, measurements in Rio de Janeiro, where ethanol was used at a level of 30% in gasoline blends, found increased levels of acetaldehyde and peroxyacetyl nitrate in the late 1980s[78,79].

#### *Catalytic Converters*

The use of MTBE has been of concern because the compound's miscibility in water has led to well water contamination across the U.S. A number of studies have shown that use of MTBE will enhance emissions of isobutene (a reactive olefin) and formaldehyde[78]. All of the oxygenated fuels will enhance aldehyde emissions during cold-start situations, before catalytic converters are warmed to operating temperatures. Use of these fuels was proposed to control carbon monoxide levels, as their use reduces carbon monoxide emissions somewhat. The oxyfuels themselves also have lower chemical reactivities (see Table 1) than alkanes, alkenes, and aromatics in gasoline. Because of these lower reactivities, the oxyfuels have been proposed as a "cleaner" fuel than gasoline. However, these claims usually do not address aldehyde emissions and are focused on the lower hydrocarbon and carbon monoxide emissions. When one considers the aldehyde and other emissions, such as enhanced $NO_x$ accompanying the use of oxygenated fuels, it becomes less clear that these fuels and fuel mixtures are as environmentally friendly as advertised. In a number of midwestern states, 10–12% ethanol-gasoline blends are currently used year round. MTBE use is being discontinued because of water contamination problems. If we are to use biomass fuels such as ethanol at higher levels than 10–12%, we will need to ensure that our catalytic converter systems are operating well enough to maintain aldehyde emissions at a low level.

#### *Health Effects*

Aldehydes photolyze to form carbon monoxide, plus hydroperoxyl and peroxy radicals, which lead to oxidant formation and increased carbon monoxide levels in urban and regional environments. Higher aldehydes such as acetaldehyde can also form formaldehyde as a product in their photooxidation reactions, as noted in earlier sections in this review. These compounds, being strong lachrymators, are known to cause eye irritation. Aldehydes, particularly formaldehyde, are suspect carcinogens and need to be viewed as separate health hazards in the consideration of alternative fuel usage and the selection of fuels[1,2,78].

### Chlorine Chemistry

Stratospheric chlorine chemistry has received considerable attention[1,2]. It may also play an important role in urban- and regional-scale chemistry[1,2]. Reactions of oxidants on the surfaces of sea salt aerosols have been shown to be a source of molecular chlorine in laboratory studies[2,80]. Field measurements have also indicated that levels of chlorine in coastal areas can be in the tens of parts per trillion[81], higher than previously anticipated for the troposphere.

#### *Chlorine Radical*

In urban environments, direct release of molecular chlorine from industrial sources leads to the formation of chlorine radical (or atomic chlorine, Cl) because of the direct photolysis of molecular chlorine by visible radiation. (Note that chlorine is a green gas.) Like hydroxyl radical, chlorine radical can react by abstraction of hydrogens from organic molecules or by addition to unsaturated sites. Chlorine radical abstraction reactions with small alkanes are much faster than analogous reactions with hydroxyl radical[1,2]. For example, chlorine reacts with ethane at a rate of $5.9 \times 10^{-11}$ $cm^3$ $molecule^{-1}$ $s^{-1}$, with propane at a rate of $1.4 \times 10^{-10}$ $cm^3$ $molecule^{-1}$ $s^{-1}$ at room temperature, and with butane at a rate of $2.2 \times 10^{-10}$ $cm^3$ $molecule^{-1}$ $s^{-1}$. Comparison with the rates for the reactions of hydroxyl radical with these low-reactivity alkanes (Table 1) indicates that reactivities with chlorine are higher by factors of 230, 120, and 90, respectively. The chlorine atom reactions approach collisional frequencies and therefore can serve as an important loss mechanism for light alkanes[1,2]. Chlorine radical reactions will yield hydrochloric acid (HCl) and peroxy radicals. This is similar to the hydroxyl radical reactions in that chlorine atoms will lead to formation of peroxy and hydroxyl radicals via the oxidation of nitric oxide.

The very high reactivity of the chlorine atoms and their formation of HCl as an end product will cause chlorine atom chemistry to be very limited spatially. Molecular chlorine will undergo photolysis in tens of minutes during summertime in most urban centers. With moderate winds of 10–20 miles per hour, the effect of molecular chlorine release in an urban area will be to enhance the nitric oxide oxidation rates via peroxy radicals formed by chlorine abstraction reactions within a few miles of the release point. This can be important, as the reactions will also enhance formation of smaller aldehydes through alkane oxidation and peroxyacetyl nitrate formation.

#### *Enhanced Oxidant Production*

The potential in the Houston area for enhanced oxidant production due to chlorine release was recently proposed as an explanation for the very high ozone levels found in relatively small pockets near heavily industrial areas[82,83]. The evidence for the presence of chlorine atoms is based upon the presence of specific chlorine adduct products formed by the reaction of chlorine atoms with isoprene[83]. We recall that tropospheric chlorine chemistry results in the formation of ozone in the presence of organics and nitrogen oxides. In the stratosphere, no organics are available, and under these conditions chlorine atoms cause ozone loss through reaction to form chlorine monoxide, ClO[1,2]. Most models do not contain this type of chemistry, which might explain high ozone levels near industrial areas that are not in attainment. The release of molecular chlorine and its local impacts should not be neglected in attempts to address urban oxidant events and determine proper mitigation strategies.

### REGIONAL AIR POLLUTION

All urban air pollution problems are reflected on regional and global scales. The key differences between urban- and regional-scale chemistry are often the levels of nitric oxide and volatile hydrocarbons emitted locally into the system. Urban levels are generally higher than suburban or regional-scale levels. As an urban plume chemically "ages," the more reactive hydrocarbons are consumed, and the less reactive hydrocarbons begin to dominate the chemistry. Similarly, on global scales the least reactive hydrocarbons begin to play a role as their reactive co-pollutants are consumed by reaction with hydroxyl radical, nitrate radical, or ozone; by photolysis; or both.

One key aspect of regional air pollution is that urban plumes of nitric oxide have often reacted to form nitrogen dioxide, peroxyacyl nitrates, nitric acid, or other nitrogen oxide end products. Thus, although nitric oxide levels might be quite high in urban areas, in regional systems the nitric oxide levels are generally much lower because of reaction and dilution. This change in nitric oxide levels affects key chemical reactions with roles in the oxidation of sulfur dioxide to sulfuric acid and also increases the importance of other aqueous-phase chemistries of hydrogen peroxide that occur on wet aerosol surfaces and in in-cloud processing of air pollutants.

The key change involves the hydroperoxyl radical. This important radical rapidly forms nitrogen dioxide and hydroxyl radical in the presence of nitric oxide, thus initiating the chain of chemistries described earlier in this paper, in the section addressing catalytic ozone formation, i.e., reactions 26–52. As nitric oxide is reduced, the hydroperoxyl radical level increases, and its reaction with itself and with peroxy radicals ($RO_2$) and peroxyacetyl radicals ($RCO_3$) results in the formation of hydrogen peroxide, organic hydroperoxides ($RO_2H$), and organic peracids ($RCO_3H$), respectively.

#### Acid Rain and Its Connections to Oxidant Chemistry

Acid rain refers to acidic precipitation observed on regional scales. Actually, rain is naturally acidic, because the carbon dioxide in air is at equilibrium with cloud water and forms carbonic acid, pH 5.6 (pH is the negative log of hydrogen ion concentration). Water is normally at pH 7, with $H^+$ and $OH^-$ concentrations equal at $10^{-7}$ M. Strong acids dissolved in rainwater can decrease the pH by increasing the hydrogen ion concentration. Organic acids can also increase the pH of precipitation. However, most organic acids, with the notable exception of oxalic acid, are weak acids and act as buffers in rainwater. Indeed, in very acidic rainwater, acids like acetic acid with a $pK_a$ of 4 can experience protonation, with subsequent volatilization and loss from the aqueous solution.

As noted in the section “Urban Air Pollution”, one of the major end products of nitric oxide and nitrogen dioxide oxidation is nitric acid. Gas-phase nitric acid, produced by the reaction of hydroxyl radical with nitrogen dioxide, is very water soluble and can be taken up by wet aerosols and cloud droplets. Sulfur dioxide reacts similarly with hydroxyl radical to form sulfuric acid, but this process is much slower than the nitrogen dioxide reaction[1,2]. Nitric acid and nitrate can also undergo photolysis to reform nitrogen dioxide and hydroxyl radical under acidic conditions in acidic aerosols or fine cloud droplets[1,2,30]. However, this photolytic source is fairly slow in the troposphere. Thus, sulfuric and nitric acids, both very strong inorganic acids, are the two important sources of acidity. As in the neutralization reactions in the gas phase, ammonia can titrate the acidity and raise the pH of precipitation. Other basic aerosols, such as carbonate, can also decrease the acidity of rain.

On regional scales, isoprene oxidation can lead to the formation of formaldehyde, methacrolein, methylvinyl ketone. These products in turn can react to form peroxy radicals and hydroperoxyl radicals. Formaldehyde, as noted earlier, can undergo photolysis to yield H atoms, with subsequent hydroperoxyl radical formation in the troposphere. In more remote (regional)

atmospheres where nitric oxide levels have been decreased, these products will in turn result in the formation of hydrogen peroxide and organic peroxides and peracids. Hydrogen peroxide is a very important oxidant for sulfur dioxide. It reacts very rapidly in wet aerosols or in cloud droplets to form sulfuric acid because of its high solubility in water (Henry's law constant of $10^5$ M $atm^{-1}$)[1,2,51]. The reaction is also acid catalyzed, which enhances the rate at higher acidities as the reaction progresses.

Thus, in areas with significant isoprene emissions, formaldehyde and hydrogen peroxide levels will be high and sulfate formation quite rapid. This expectation is consistent with studies of the aerosol composition in the Great Smoky Mountains, where high aerosol acidity occurs during the summer months, when deciduous trees are very active[1,2,51,84,85], and where the predominant summertime aerosol component is sulfate. Polluted precipitation, which continues to be a problem on regional scales, involves acids and other soluble species including hydrogen peroxide and organic peracids, as well as nitrophenols and other potent phytotoxins[1,2,51]. Thus, the oxidized products of the organics that are water soluble undergo wet deposition and should be included in considerations of regional impacts of air pollution, in addition to acidic species.

### Ozone and Peroxyacyl Nitrates

Regional-scale formation of ozone and peroxyacyl nitrates can also be important. As noted earlier, the peroxyacyl nitrates are in thermal equilibrium with the peroxyacetyl radical and nitrogen dioxide. The decomposition of peroxyacyl nitrates will lead to the production of peroxy radicals and nitrogen dioxide, which in turn can oxidize nitric oxide to form nitrogen dioxide and then increase ozone levels as nitrogen dioxide is photolyzed. Regional ozone levels appear to be increasing, while urban-area levels have been decreasing. This is due in part to the decrease of reactive hydrocarbon emissions from automobiles and stationary sources in the urban areas. Application of catalytic converters to reduce carbon monoxide and hydrocarbon emissions from the mobile sources has been quite successful. However, nitric oxide and nitrogen oxide ($NO_x$) emissions have not decreased. The result is situations where the natural hydrocarbons, particularly isoprene, can play an important role in the production of ozone and peroxyacyl nitrates. For example, Nashville, Tennessee, has increased levels of ozone that are due to isoprene influences from the surrounding forested areas[86,87].

As the lower-reactivity organics continue to react with hydroxyl radical, they are dispersed from the urban scale to the regional scale, and the formation of ozone, peroxyacyl nitrates, and other products continues over longer time periods during the transport and dispersion of the urban plumes. Thus, the emission of volatile organics with nitrogen oxides will continue to lead to formation of ozone and other oxidants, acids, etc. as long as there is appreciable $NO_x$ in the air. Only when the nitric oxide levels are low will reactions with the hydroperoxyl radical begin to remove the odd oxygen species via loss of hydrogen peroxide or reaction of the hydroperoxyl radical with itself or other peroxy radicals.

### Long-Range Transport of Gases and Aerosols

For many years, the "Los Angeles smog" chemistry was assumed to be found only in this urban environment. Now it is clear that this chemistry is key in the overall chemical processing of many gases, both natural and anthropogenic in origin. The reactions of hydroxyl radical, ozone, nitrate radical, etc. are occurring on all scales. The reactivity of the organics and their transport and dispersion differentiate the various levels of pollutants observed in urban-, regional-, and global-scale environments. Long-range transport of gases and aerosols is now very well documented.

Recent applications of satellite imagery have allowed the direct observation of large-scale events. One example was the Mexican brush fires that occurred over a very large scale in the summer of 1999. Smoke plumes were observed as far away as Canada before they dispersed to levels that were not observable optically. During that period we observed low-level transport that affected Phoenix, Arizona, resulting in reduced visibility, actinic radiation, and subsequent reduction in the photolysis of nitrogen dioxide and in ozone formation[88].

### *Transported Gases*

Observations of high levels of ozone, peroxyacyl nitrates, and other oxidants at night are clear indicators of long-range transport of these pollutants. Such observations and increasing background levels of ozone, peroxyacyl nitrates, and aerosols all are indications of long-range transport. As we continue to add combustion-related pollutants to the atmosphere, the old adage, "the solution to pollution is dilution," is clearly not valid. We live in one atmosphere, and the pollution emitted into it can have impacts over long distances. This is particularly the case for pollutants with longer lifetimes, specifically the organics with lower reactivity. These pollutants can be transported and distributed over very wide areas. The best example of this is the essentially tropospherically inert chlorofluorocarbons. These greenhouse gases, when transported into the stratosphere, lead to ozone destruction as they are photolyzed or react to form chlorine radicals.

### *Transported Aerosols*

Fine aerosols can also be transported long distances. Large aerosol particles are removed by gravitational settling. Ultrafine aerosols tend to diffuse to existing aerosol surfaces, but fine aerosols with aerodynamic diameters of 0.1–1 μm have considerable residence times[1,2]. The hydrophilic aerosols are removed by wet deposition and removal, and thus sulfate and nitrate are washed out of the systems rapidly. However, even these water-soluble species probably have residence times on the order of 10 days, on the basis of observations of natural radioactive tracers in the atmosphere[89]. The lifetimes of other hydrophobic aerosols such as carbonaceous soots are likely to be longer. Estimates of aerosol residence times for fine aerosols, obtained by using $^{210}Pb$ and its daughters, indicated lifetimes of 20–80 days for some fine aerosol particles[90,91,92]. These results are not inconsistent with observations of arctic haze and large dust storm impacts due to finer fractions crossing the Pacific from the Gobi Desert and crossing the Atlantic from the Sahara.

### *Transformations during Transport*

It is important to recognize the importance of long-range dispersion and transformation during transport. By reducing the reactivity of emissions to decrease urban air pollution but we have sometimes increased regional pollution and ozone and aerosol backgrounds in other areas. Our past considerations of "air shed" control strategies need to be reevaluated. Emissions must be controlled at the source to eliminate the longer-range impacts of airborne pollutants.

### GLOBAL-SCALE IMPACTS

As noted just above, urban emissions lead to regional- and global-scale problems. The examples presented here are not meant to be all-inclusive but rather to show how air pollutants and their larger-scale impacts are interconnected. Most recent studies of global-scale impacts have focused on climate change issues, addressing the potential for combustion-related emissions to cause global warming (or cooling). Most of the literature has addressed carbon dioxide and some of the trace greenhouse gases. Sulfate aerosol has been examined as a light scatterer and cooling agent[1,2]. The focus has been principally on infrared-absorbing gases and shortwave-scattering or -absorbing aerosols.

#### Greenhouse Gases

In principle, any gas that has infrared absorption bands will act as a greenhouse gas. The best known are carbon dioxide and water vapor. However, a number of other species are also important infrared absorbers. These include longer-lived species in the troposphere such as chlorofluorocarbons, methane, and nitrous oxide, which are considered trace greenhouse gas species. These actually can be very important, because many of them have strong bands in window regions where carbon dioxide and water vapor do not absorb outgoing infrared radiation[1,2].

#### Other Gases and Radiative Balance

Many of the other air pollutants can also be important in absorbing or scattering incoming shortwave radiation or absorbing outgoing longwave radiation[1,2,93]. Infrared-active gases include ozone, nitrogen dioxide, peroxyacetyl nitrate, carbon monoxide, formaldehyde, ethene, and many organic compounds. Indeed, as noted, the increased levels of these gases, occurring daily with higher levels of carbon dioxide due to combustion of fossil fuels in urban areas, can lead to urban heat island effects. The increases in urban temperatures can be substantial and need to be considered in considerations of overall effects of air pollutants.

#### Aerosols and Radiative Balance

Aerosols, as noted earlier, can lead to scattering and cooling [1,2,93,94]. Carbonaceous soots formed by combustion processes can also act directly to absorb incoming shortwave radiation and thus heat the troposphere. Black carbon has a continuously level absorption band strength across the visible region, making it a very effective radiative material[95]. In polluted environments, carbonaceous soots are essentially the only visible-light-absorbing materials in the air. The inorganic aerosols such as ammonium sulfate or ammonium nitrate are white and scatter light very efficiently. Both the scattering aerosols and the absorbing aerosols contribute to visibility loss and to radiative balance impacts in incoming radiation fields. Furthermore, these same aerosols also have strong infrared absorption cross sections and might affect local heating of air during both daytime and nighttime[93,94].

Direct heating by carbonaceous soots has been addressed by using global-scale modeling. The results indicate significant impact by soots in the Northern Hemisphere. Indeed, because they have a much shorter lifetime than carbon dioxide, control of carbonaceous soots could be the most effective way to slow global warming of all the control strategies currently being examined[96].

Aerosols can also have indirect effects on radiative balance. Many hydroscopic aerosols, such as sulfate and nitrate, can act as cloud condensation nuclei. Increases in these nuclei can increase cloud formation and subsequent light scattering or absorption by liquid water, another important greenhouse species[97]. Thus, depending on its type and its position in the atmosphere, a cloud can cool or heat the atmosphere. Long-range transport of aerosols and their subsequent interactions must be considered when we address radiative balance and the associated effects on precipitation, because aerosols are important in the global hydrologic processes that are tied to climate change.

### SOME FINAL COMMENTS

Air pollution chemistry is now recognized as being critically important on all scales (urban, regional, and global). Since the earliest investigations, human industrial and energy-related activities (especially fossil fuel combustion) have been recognized as likely causes of observed effects on health and agriculture. As we continue to examine the chemistry of trace gas species, we now realize that the reactions that lead to high levels of pollution under urban and regional conditions are also important means of cleansing the atmosphere of many toxic trace gas species. Although hydroxyl radical reactions can lead to harmful increases in ozone in the troposphere, the same reactions also remove methane and other organics that would have deleterious effects in the stratosphere and troposphere if they were allowed to accumulate[1,2]. Thus, trace gas chemistry has a balance that needs to be appreciated. For example, although ozone levels above 60 ppb are almost always detrimental, a background level is needed for continuing oxidation and removal of trace gas species generated naturally and by human activities. Only when we understand the chemical and physical processes involving trace gases and aerosols, as well as their interactions, will we be able to plan pollution control and energy usage strategies that lead to environmental sound practices on all scales.

This review is meant to give the reader a brief background on some key chemical processes and species involved in air pollution. This subject area has grown considerably in the 60 years since the first observations of photochemical air pollution in Los Angeles in the 1940s. The reader is referred to recent books on the subject for further detailed information[1,2]. We are just beginning to understand the significance of our atmosphere and our impacts on it. Air is a precious resource that needs to be understood and guarded. The hope is that continued work in atmospheric chemistry and physics will build the knowledge base needed for informed policy decisions that will safeguard future generations.

### ACKNOWLEDGMENTS

The authors wish to acknowledge the continuing support of the Atmospheric Science Program, Environmental Sciences Division, Office of Biological and Environmental Research, Office of Science, U.S. Department of Energy. We also wish to thank Ms. Virginia Manners for reviewing the manuscript and for helpful suggestions, and Ms. Karen Haugen her skilled work in editing this mini-review.
